# The Five Immune Forces Impacting DNA-Based Cancer Immunotherapeutic Strategy

**DOI:** 10.3390/ijms18030650

**Published:** 2017-03-17

**Authors:** Suneetha Amara, Venkataswarup Tiriveedhi

**Affiliations:** 1 Department of Medicine, St. Thomas Health Mid-Town Hospital, Nashville, TN 37236, USA; suneetha.amara@sth.org; 2 Department of Biological Sciences, Tennessee State University, 3500 John A Merritt Blvd, Nashville, TN 37209, USA

**Keywords:** DNA vaccine, T-cells, cytokines, immune checkpoint inhibitors, tumor associated antigens

## Abstract

DNA-based vaccine strategy is increasingly realized as a viable cancer treatment approach. Strategies to enhance immunogenicity utilizing tumor associated antigens have been investigated in several pre-clinical and clinical studies. The promising outcomes of these studies have suggested that DNA-based vaccines induce potent T-cell effector responses and at the same time cause only minimal side-effects to cancer patients. However, the immune evasive tumor microenvironment is still an important hindrance to a long-term vaccine success. Several options are currently under various stages of study to overcome immune inhibitory effect in tumor microenvironment. Some of these approaches include, but are not limited to, identification of neoantigens, mutanome studies, designing fusion plasmids, vaccine adjuvant modifications, and co-treatment with immune-checkpoint inhibitors. In this review, we follow a Porter’s analysis analogy, otherwise commonly used in business models, to analyze various immune-forces that determine the potential success and sustainable positive outcomes following DNA vaccination using non-viral tumor associated antigens in treatment against cancer.

## 1. Introduction

Advances in immune understanding have enhanced optimism towards DNA-based vaccine therapies against cancer. While the traditional treatment approaches such as tumor resection, radiotherapy, and anti-cancer chemotherapy have shown success in early stage localized tumors, they have only limited role against later staged metastatic malignancies. Furthermore, these standard agents have shown to cause extensive damage to normal tissues leading to hair loss, blood cell destruction, and debilitating side effects such as decreased appetite, hair loss, and immune-suppression. All of these lead to more dangerous secondary infections in these patients. Thus, immune-based therapeutic strategies offer viable long-tern success by specifically eliminating cancer cells and inducing relapse-free quality survival in cancer patients [1].

Recently, DNA-based vaccines have been developed as a concrete and viable approach and anti-cancer treatment strategy [2]. Advances in the field of recombinant plasmid technology have significantly reduced the costs in the vaccine preparation. Further, high-throughput tools have been developed to quickly identify tumor associated antigens and thus providing the required target genes for plasmid design. This design of recombinant plasmid backbone requires incorporation of gene coding for specific tumor associated antigens (TAA) with a potential to induce the desired immune effector response, and an inducer of T-cell help for induction of durable immune memory through efficient antigen expression and presentation while at the same time evading immunosuppression [3]. The three major aspects of DNA vaccination strategy include critical plasmid design, efficient delivery, and specific post-vaccine immune-monitoring tests.

DNA vaccines have a promising application both in the prevention of cancer and also in the treatment of already existing cancers. The ability of DNA-based vaccines to mount an effector T-cell-based immune responses make them an attractive anti-cancer therapeutic tools. For example, a major breakthrough was achieved in the prevention of cervical cancer with recombinant protein-based human papilloma virus (HPV) vaccination, as HPV (strains 16 and 18) have been strongly correlated with the development of cervical cancer. This approach has demonstrated an upregulation of humoral (B-cell based) immune responses as evidenced by increased titers of anti-capsid antibodies that could neutralize HPV capsid and prevent the development of HPV infection. However, this approach did not mount an efficient cellular (or T-cell-based) immune response. While efficient prevention of cervical cancer with HPV vaccination was effected through induction of potent antibody response, the vaccine did not demonstrate significant protective effect on already infected patients since they had already undergone antigen-expression changes in infected cells [4]. Therefore, for the prevention of cervical cancers in patients with persistent/previous HPV infections, it is critical to activate effector T-cell responses against major histocompatabilty complex (MHC)-bound peptides derived from antigens specific to these disease states which can be efficiently achieved by DNA-based vaccines over protein-based vaccines.

**Figure 1:**
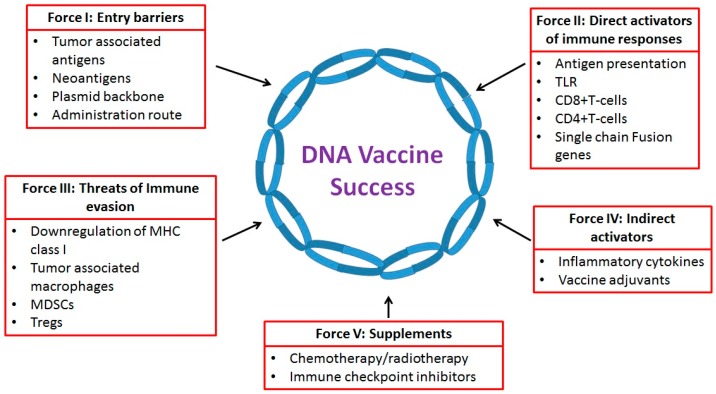
The five forces immune framework affecting DNA vaccine outcomes in cancer therapy. MHC, major histocompatibility complex; TLR, toll-like receptor; MDSCs, myeloid-derived suppressor cells.

Currently there are 340 clinical trials on cancer DNA vaccines that are either actively recruiting or have approval for open recruiting (www.clinicaltrials.gov, accessed on 17 January 2017). The long-term success of DNA vaccines as an efficient anti-cancer tools is dependent upon multiple immunological factors. To comprehensively analyze the impact of multiple immune-factors, in this review, we adopt a Porter’s strategy analysis analogy to discuss specific immunological factors that affect the potential success of this novel anti-cancer therapeutic strategy. The Porter’s model is a standard method to analysis various factors affecting the success of a new business [5]. Here, we borrow the Porter’s analogy to discuss the potential immune-forces (Figure 1) affecting the success of DNA-based immunotherapeutic strategy against cancer. This Porter’s method is considered one of the best analysis strategies that allows us to approach a scientific problem both at a macro- and micro-level, so that a problem is analyzed for its realistic current standing, future challenges, potential strengths, while at the same time it defends against the threat of failure, and specifically in our case, helps us develop an effective long-term successful DNA-based vaccine strategy. While the specific five force nomenclature in Porter’s model is geared to approach business-oriented challenges, we have made adequate changes to suit the approach and challenges of life sciences, specifically anti-cancer DNA vaccines.

## 2. Force I: Entry Barriers—Target Choice and Delivery Techniques

A judicious choice of antigen and the method of entry into the human body are clearly the first step towards successful DNA vaccine design and administration. Antigens expressed in cancers of non-viral etiology are attractive targets for DNA-based vaccines as these antigens can mount an effector immune response specifically against cancer cells [6]. These cancer specific antigens are traditionally called tumor associated antigens (TAA). Another group of cancer specific antigens arising from somatic mutations in cancer cells are called neo-antigens. Further, there are a few tumor-specific antigens with idiotypic immunoglobulin of B-cell, malignancies being a noteworthy exception. Antigen is expressed on normal cells or tissues as a part of normal development of tissue or cell differentiation, as is the case in lineage-specific antigens, which fail to induce a strong effector immune response and so are generally considered a poor antigen choice for DNA vaccine development. Furthermore, it is extremely important that the newly developed DNA vaccine should cause minimal immune-related damage to normal terminally differentiated cells/tissues and if at all should cause only nonlethal localized side effects such as a temporary rash at the site of injection. In several pre-clinical and clinical trials, these TAA-based vaccines have demonstrated encouraging therapeutic benefits, importantly, by extending the over-all disease-free survival in cancer patients [7].

### 2.1. Tumor-Associated Antigens

DNA-based cancer vaccine development has utilized different types of tumor-associated antigen (TAA) primarily depending upon the solid organ and cell-type from where the cancer originates. The TAAs are either expressed in the tumor tissue, overexpressed by oncogenes or selected as differentiation antigens during cancer development. In 2009, the National Cancer Institute (NCI) has published 75 cancer antigens that are used in developing DNA-based vaccines [8]. Unfortunately, the T-cell effector responses against several of these TAAs can be diminished by central tolerance. Further, various studies have demonstrated that the vaccine activated T-cells have limited ability to induce tumor cell destruction mainly due to the pre-existing immune-suppressive tumor microenvironment [9]. Another important concern that researchers should be aware of is that DNA-based vaccines are dependent on the T-cell repertoire left behind following depletion of the high and low-affinity T-cells, but retained medium-affinity T-cells during the early T-cell development stage in spleen and lymph nodes [10]. Thus, these retained medium-affinity T-cells many not sufficiently activated to high frequency following DNA vaccine. To circumvent this drawback, a combination DNA vaccine strategy such as PROSTVAC-VF could be utilized, where in two recombinant viral vectors were used. Each vector encodes for a TAA and three T-cell costimulatory molecules such that one is used for initial priming and the other is for booster effect [11]. Such innovative approaches could ensure a more viable immune response against the TAAs. A list of some of the currently utilized TAAs in DNA vaccination are listed in Table 1.

In our studies, we have identified mammaglobin-A (Mam-A), a breast cancer specific TAA, as a viable option to induce immune responses following DNA vaccination in breast cancer patients. Previous dendritic cells–based studies with TAAs, human epidermal growth factor-2 (HER2/neu), andmucin-1 (MUC1) have demonstrated expansion of peptide-specific CD8+ cytotoxic T-lymphocytes (CTLs) in breast cancer patients [7,26]. However, the lower frequency of expression of these TAAs (HER2/neu: 20%–30% and MUC1: up to 60%) on breast tumors limits a broader application of these TAAs as a viable immunotherapeutic strategy [27,28]. In contrast, the Mam-A, a 10 kDa glycoprotein, is expressed on more than 80% of breast tumors across all individual breast cancer types and stages [18,29]. Further, Mam-A is shown to have exclusive expression on breast cancer cells, with virtually no expression on other tissues, thus making it a uniquely specific marker for detection of breast cancer cell metastasis to draining lymph nodes as compared to other markers (such as HER2/neu and cytokeratin-19) [30]. Pre-clinical murine studies have demonstrated that passive transfer of T-cells from Mam-A vaccinated human leukocyte antigen (HLA)-A2/hCD8 double transgenic mice into a human breast cancer implanted NOD/SCID mouse resulted in significant tumor regression [31,32,33]. Based on these encouraging pre-clinical studies, we instituted a phase I clinical trial of a Mam-A cDNA vaccination in breast cancer patients with metastatic disease. Our studies demonstrated that following Mam-A cDNA vaccination, there was an upregulation of tumor lytic CD4+ ICOS^hi^ T-cells in Mam-A vaccinated breast cancer patients [34] and also induction of antigen specific CD8+ T-cell effector responses [18].

### 2.2. Neoantigens

Theoretically, the most potent antigen to be used in the development of DNA vaccine would be completely non-native to the patient, so that there is no pre-existing central tolerance. Neo-antigens are the self-antigens that mutate to form novel non-self-antigens [35]. The mutations could be induced by a variety of external and environmental factors such as carcinogens, UV light etc. [36]. Initial evidence of vaccination with neo-antigens was found in the B16 mouse melanoma model, wherein vaccination against two synthetic mutant antigens resulted in a marked tumor regression [37]. Studies on metastatic melanoma patients have demonstrated that treatment with anti-cytotoxic T-lymphocyte associated protein-4 (CTLA-4) mAbs was more effective in patients with higher mutational load, thus suggesting that an upregulation of effector immune responses to neoantigens following anti-CTLA-4 therapy [38]. Further, various studies have shown that tumor infiltrating lymphocytes (TILs) with higher tumor regression capability constituted of CD8+ and/or CD4+ T-cells specifically against neoantigens [39]. However, the research on neoantigens and mutanome analysis is still infancy, and it would be interesting to the future outcomes of this research.

### 2.3. Plasmid Backbone

**Figure 2:**
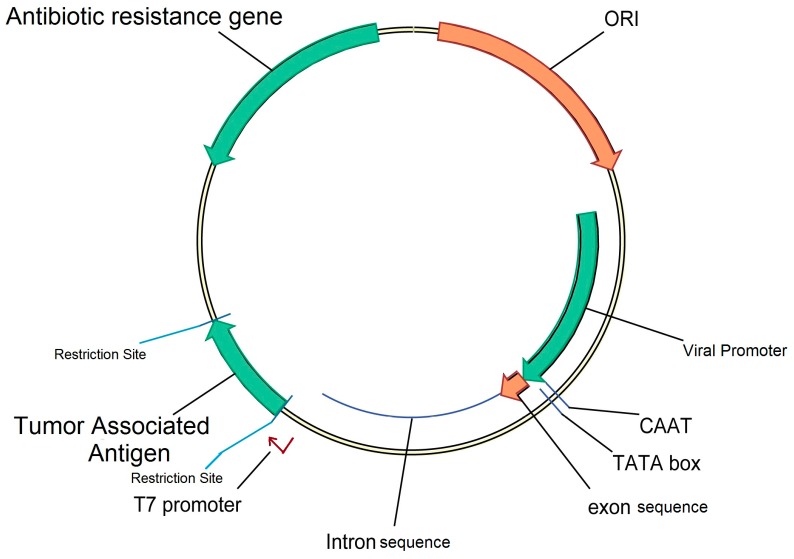
Generic structure of the plasmid backbone for DNA Vaccine. ORI, origin of replication; Colors represented in green are usually inserted into the vector backbone; color represented in green usually are already present in the vector backbone.

Important characteristics of a DNA vaccine plasmid backbone would include a strong promoter sequence, an antibiotic selection marker, and a poly-A sequence to stabilize the mRNA transcript (Figure 2). The traditionally used promoter for human DNA vaccines is human CMV promoter, as it induces higher expression of the target gene in a wide array of tissues and at the same time does not suppress downstream read through [40]. Recently, the incorporation of HTLV-1R-U5 downstream of the CMV promoter or chimeric SV40-CMV promoter has been shown to enhance vaccine efficiency [41]. Further, target gene expression can be enhanced by the addition of an intron in the vector backbone and by the introduction of kozak sequence immediately upstream of the gene of interest’s start codon [42]. Gene expression can be manipulated by altering the polyA sequence, which is required for proper termination of transcription and export of mRNA from the nucleus. The plasmid backbone is thought to stimulate innate immunity via specific CpG dinucleotide repeats. This pathway involves uptake of CpG-rich DNA vector via receptor for advanced glycated end products (RAGE) and signaling through endosomal toll like receptor TLR-9/MyD88 to induce type I interferon response [43]. However, TLR9 double knockout mice have demonstrated equivalent immune responses compared to wild type mice. This evidence potentially suggests that multiple cytoplasmic pattern recognition receptors act as sensors for plasmid DNA vector, which might include factors such as DNA dependent activator of IFN-regulatory factor (DAI), retinoic acid inducible protein-1 (RIG-1) and helicases, which lead to expression of other pro-inflammatory transcription factors [44]. Further, DAI co-delivery with melanoma antigen in a DNA vaccine has shown enhanced pre-clinical efficiency [45]. The AT rich regions in the plasmid backbone is associated with nicking leading to open circular plasmid and is amenable to endogenous nucleases [46]. Instability in the final plasmid construct could be caused by palindrome sequences, direct or inverted repeats. Specifically direct repeats in the plasmid are considered hot spots for mutations [47]. Plasmids containing Z-form DNA (left handed double helical structure, as against the more common B-form, right handed double structure) regions are unstable, possibly due to the formation of triplex regions due to endogenous nuclease [48,49].

### 2.4. Administration Site

DNA vaccine should be administered with an aim to deliver the TAA gene to the dendritic cells so that there will be efficient antigen presentation. This is effectively achieved when DNA vaccine is delivered by intramuscular (i.m.) route. However, the efficient T-cell activation will depend on the dendritic cell’s homing ability to the nearest lymph node. Recent evidence suggests that mucosal cancers are more efficiently treated when vaccine-activated T-cells can home-in to mucosa-associated lymph nodes, thus suggesting alternate routes (such as intra nasal) as viable options [50]. Other DNA vaccine delivery methods such as particle mediated gene guns [51], needle-free systems [52], liposomal coating [53], and mucosal delivery [54] are under various stages of study. However, the variability of human immune response signaling following these delivery strategies is still unclear. Much more research is needed to determine the precise rules of human cancer vaccine design specific to various routes of administration.

## 3. Force II: Direct Activators of T-Cell Responses

### 3.1. Role of Antigen Presenting Cells

DNA vaccine design is done such that there is concentrated antigen delivery to dendritic cells [55]. These activated dendritic cells induce upregulation of both CD4+ and CD8+ T-cell responses. The CD4+ T-cells are needed for optimal and sustained effector and cellular memory responses. Direct loading of dendritic cells with the HLA-binding epitopes, as is the case with Provenge, has met with limited success [56]. Further, dendritic cell vaccines proved to be extremely expensive as they have to be acquired from the tumors of the patients and cultured in vitro for six weeks before they are primed and re-injected into the patient, thus warranting further research and optimization for better manufacturing protocols.

### 3.2. Role of CD8+ T-Cells

Following administration of a DNA vaccination, the expressed TAAs, when presented in the context of MHC class I proteins, induce activation of CD8+ T-cells. These newly activated CD8 T-cells, upon contact with tumor cells, exert their effector function by lysing the tumor cells through the release of pore-forming perforin in a calcium dependent manner onto the target cell membrane [57]. The activated CD8+ T-cells also release high levels of inflammatory cytokines, such as interferon-γ (IFNγ) and tumor necrosis factor-α (TNFα), to induce a conducive environment for anti-tumor response. However, the exact cells involved in the activation of CD8+ T-cells following vaccination is still debated, as muscle cells express negligible amounts of MHC and other co-stimulatory molecules rendering muscle cells as inefficient antigen presenting cells. Thus the professional antigen presenting cells, dendritic cells and macrophages, in the muscular tissue might still be needed for activation of CD8+ T-cell following DNA vaccination [58].

**Figure 3:**
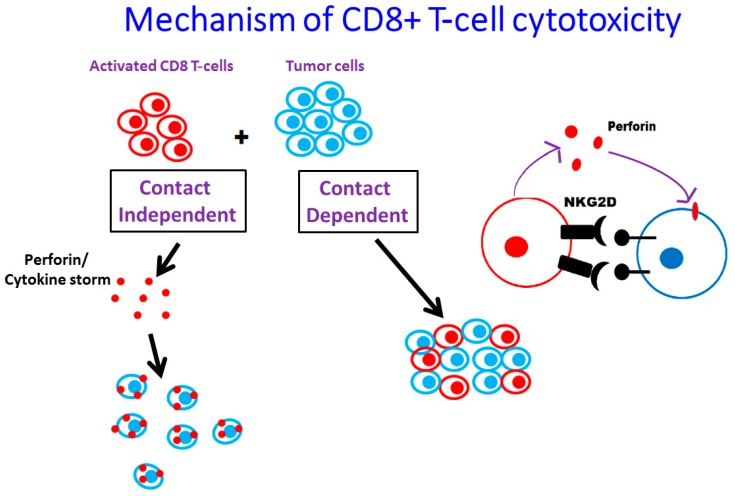
Contact dependent T-cell mediated tumor cell death. The T-cells activated following DNA vaccination were checked for antigen specific response. Our studies demonstrated that inflammatory release from Mam-A activated CD8+ T-cells is specifically upon contact with target tumor cell, and there by avoids a generalized cytokine storm response following vaccination.

Our studies with the Mam-A DNA vaccine, utilizing a tetramer-based assay approach, have demonstrated that Mam-A cDNA vaccination to HLA-A2 patients specifically expanded the CD8+ T-cells specific to HLA-A2 immunodominant epitope of Mam-A (LIYDSSLCDL). The effector response of these activated CD8 T-cells has been thought be mediated by the inflammatory cytokines (IFN-γ and TNF-α) [59] and also by the secretion of pore forming protein, perforin [60], ultimately leading to lysis of the target cell. Our data demonstrates that Mam-A DNA vaccination in advanced breast cancer patients induced activation of CD8 T-cells and upregulation of the intracellular expression of all these three effector molecules, namely, IFNγ, TNFα, and perforin. Natural killer group 2D (NKG2D), an activating cell surface receptor, expression was significantly correlated with IFN-γ production in CD8+ T-cells in the human melanoma studies [61]. Our studies have demonstrated that following MamA cDNA vaccination, there is MamA specific upregulation of NKG2D expression, which is induced by the inflammatory cytokines IFN-γ and TNF-α. Further it is important to note the inhibition of NKG2D expression by specific NKG2D antibody thus, suggesting direct cell-contact dependent cytotoxic NKG2D signaling [18]. We have further demonstrated that this NKG2D engagement by vaccine activated CD8+ T-cells is tumor cell contact dependent, thereby avoiding a generalized cytokine storm which is a common cause of vaccine failure (Figure 3).

In humans, NKG2D has been shown to exert its signaling through association with the adaptor molecule DNAX-activation protein 10 (DAP-10) [62]. Furthermore, expression of DAP-10 has been correlated with interleukin (IL)-2 induced NKG2D mediated cytotoxicity in CD8+ T-cells. Our studies have shown that CD8+ T-cells from Mam-A vaccinated HLA-A2+ breast cancer patients induced expression of DAP-10 leading to NKG2D mediated contact dependent cytotoxicity of tumor cell lines. Interestingly, albeit as expected, siRNA and antibody inhibition of NKG2D and DAP10 signaling inhibited the release of pore-forming perforin molecules from CD8+ T-cells [18].

### 3.3. Role of CD4+ T-Cells

The CD4+ T-helper cells, which are predominantly activated by epitopes in the context of MHC class II molecules, have a major role in production of antibodies by B cells and induction of memory CD8+ T-cell responses. It is also known that a subset (5%–15%) of CD4+ T-cells can act as regulatory cells (Treg), which specifically in the context of cancer unfortunately promotes cancer growth [63]. Therefore, ideally, vaccination induced activation should specifically activate helper and effector CD4+ T helper response while suppressing Treg response. Along with helper response of CD4+ T-cells, several studies [64,65] have demonstrated an effector cytotoxic effector response for CD4+ T-cells. Mainly, this cytotoxic phenotype of CD4+ T-helper subsets (Th1/IFN-γ) correlates with inducible co-stimulatory molecule (ICOS) expression [66]. The ICOS expression has been correlated with newly activated CD4+ T-cells [67]. The higher frequency of regulatory T-cells (FoxP3+ CD4+ T-cells, Treg) that have been seen in melanoma [68] prostate [69], and bladder cancers [70] were implicated in evasion of immune system favoring continued growth of the tumors. Therefore, vaccine success can be theoretically determined by the changes in the ratio of the ICOS (anti-tumor effector activity) to FoxP3 (pro-tumor immunosuppressive activity) expression on CD4+ T-cells. In our studies on breast cancer patients, we have demonstrated that FoxP3+ Treg frequency is 2–3 times higher in breast cancer patients (19% vs. 7%) over normal subjects. However, following Mam-A vaccination, there was a decreases in the Treg frequency (19% vs. 10%) in the cancer patients’ pre- (19%) and post- (10%) vaccination. Although not statistically significant, which might be due to small number of patients (*n* = 8) analyzed in our study. Further, in the same cohort, the ICOS expression on CD4+ T-cells remained constant (approximately 21%) in breast cancer patients prior to and following vaccination similar to that seen in normal subjects. However, interestingly, the ratio of ICOS+ CD4+ T-cells to FoxP3+ CD4+ T-cells significantly increased from 7% pre-vaccination to 23% post-vaccination, which is also accompanied by a three-fold increase in the IFN-γ response in CD4+ T-cells following vaccination to breast cancer patients. This data strongly suggests that following Mam-A cDNA vaccination there is increased activation of anti-tumor CD4+ T-cells [22]. In nutshell, our studies clearly suggest that Mam A DNA vaccination has a strong therapeutic application in breast cancer patients, which however needs to be further confirmation in a large multi-center trial.

### 3.4. Role of Fusion Genes on T-Cell Activation

To induce activation of CD4+ T-cells from the non-tolerized CD4+ T-cells naïve repertoire, several research groups have fused the variable region genes of the immunoglobulin called single-chain Fv (scFv) sequence with the TAA gene in the recombinant plasmid [71]. As previously mentioned medium-affinity CD4+ T-cell repertoire is already established during development of central tolerance, engaging a new repertoire of CD4+ T-cells could overcome this challenge of central tolerance. Studies by Rice et al. have shown that it is critical to deliver CD4+ T-cell epitopes to the same professional antigen presenting cells (APCs) that can express tumor antigens to promote stimulation of specific antitumor CD4+ T-cell idiotype [72]. The mechanism of priming APCs could done by the scFV fusion plasmid. Further, these fusion genes have also been shown to stimulate expression of co-stimulatory molecules such as B7.1, CD28, and OX-40 following recombinant plasmid injection to favor anti-tumor CD4+ T-cell activation [73]. Further, this scFv fusion gene approach will help the maintenance of antigen-presenting function by dendritic cells and thereby probably allowing utilization of otherwise weak TAAs in the DNA vaccine development. Further, several researchers have shown the utility of the full length tetanus toxin fragment C protein (Frc), with known promiscuous epitopes that widely bind with murine and human MHC class II molecules to induce viable anti-tumor CD4+ T-cell response. However, when the full-length FrC gene sequence is replaced by small epitope sequence in the recombinant DNA vaccine generation, there was a significant (>80%) reduction in the vaccine efficacy [74]. Taken together, all these exciting preliminary studies in the scFv recombinant plasmid technology requires further research and development for clinical application.

## 4. Force III: The Threat of Immune Evasion

### 4.1. Downregulation of MHC Class I on Tumor Cell

As previously discussed MHC class I molecules, expressed on all nucleated cells, play crucial role in the antigen presentation and activation by cytotoxic CD8+ T-cells. Several studies have shown that specifically in the tumor there is decreased cell surface expression of MHC class I proteins [75]. Along with the specific decrease in the MHC class I protein expression, the transcript levels of several other proteins required in the class I presentation pathway were also decreased in tumor cells. Importantly, the mRNA transcript levels of endoplasmic reticulum associated transporter protein, TAP, which is critical to antigen processing (TAP), were reduced following transfection of the cells with *HRAS* oncogene, suggesting a direct correlation between cancer cell transformation and downregulation of antigen presentation pathway. A consequential phenomenon of lowered vaccine induced CD8+ T-cell activation was observed against *HER2* oncogene expressing breast cancers [76].

Recently, a subset of CD8+ T-cell were identified that possess a capability to recognize and eradicate cancers with impaired antigen presentation machinery (APM). Interestingly, these CD8+ T-cell’s immune detection of APM deficient cells is linked to T-cell receptor (TCR) and not natural killer (NK) cell receptors. The TAAs recognized by these TCR were shown to have been immaturely process as so these unique epitopes of are called TEIPP, T-cell epitopes associated with impaired peptide processing [77]. The TEIPP antigens are considered to originate from wild-type sequences of housekeeping genes such as TRAM-protein homolog 4. Further as TEIPP antigens are processed through non-classical antigen presentation pathway it is assumed there is no central tolerance against these antigens, thus making these TEIPP antigens an extremely interesting target for future DNA vaccine development.

### 4.2. Tumor Associated Macrophages

Research evidence has established that the tumor microenvironment has a higher concentration of immune-suppressive, pro-tumor, alternatively activated M2 macrophages. These M2-type macrophages participate in TH2 cell responses, dampen inflammation, suppress immunity, and promote tumor progression [78]. A strong infiltration of M2-type macrophages is associated with poor prognosis in many tumors. Further, following tumor resection surgery, a quick reconstitution of residual tumor with M2-phenotype macrophages has shown to promote tumor regrowth and thus probably also limit the success of DNA vaccination [79].

### 4.3. Myeloid-Derived Suppressor Cells (MDSCs)

The immature myeloid cells called MDSCs are found in large numbers in the blood and tumors of patients with cancer. These tumor localization of MDSCs is favored by the production of cytokine GM-CSF by tumor cells [80]. These tumor associated MDSCs inhibit the potential anti-cancer maturation and activation of naive T-cells within the tumor microenvironment. Pre-clinical animal models have demonstrated that MDSCs hinder vaccine-induced CD4 and CD8 T-cell responses. A direct negative correlation was demonstrated between circulating MDSCs and patient survival following anti-CTLA4 treatment in melanoma patients [81] and patients who received immunotherapeutic vaccination in small cell lung cancer [82].

### 4.4. Regulatory T-Cells (Tregs)

The Tregs are a subset of CD4+ T-cells with immune-suppressive cell type present in the tumor microenvironment. Furthermore, Tregs have been shown to exert there immune-suppressive effect both in tumor-specific and tumor-nonspecific manner [83]. Several studies have demonstrated a negative correlation between Treg frequency and tumor prognosis. Also, in the context of anti-cancer vaccination, pre-clinical and clinical studies indicate worse vaccination outcomes when there is high frequency of Tregs [84].

## 5. Force IV: Indirect Activators

### 5.1. Inflammatory Cytokines

The cytokines, Type I interferons (IFNs) and interleukin-12 (IL-12) are required for an optimal CD8+ T-cell activation and effector cytotoxic responses. These inflammatory cytokines are produced by both innate and adaptive immune cells. However, the local concentration and kinetics of the cytokine release depend on the nature of immune cell activation and the type of cells activated during immune response [85]. IL-12 has been shown to activate T-cells, whereas type I IFNs are known to directly stimulation of CD8+ T-cells and other cell types, including APCs. Therefore, several researchers have combined these inflammatory cytokines in their DNA vaccine strategies to enhance anti-tumor response [86]. Similarly, IL-2 is another pluripotent cytokine of interest that has been shown to induce T-cell maturation and proliferation [87]. Future cytokine combination to DNA vaccination strategies could offer higher theoretical success, but would require several large studies to confirm this idea.

### 5.2. Adjuvants

A critical pre-requisite for successful DNA vaccination strategy is the correct choice and dose of vaccine adjuvant. The adjuvants work by induction of costimulatory molecules such as toll like receptor factors (TLR 3 and 9) through binding with its ligands such as poly I:C and CpG islands on cellular DNA [88]. Just like scFv fusion strategy mentioned above, DNA vaccines can be further improved by gene fusing with TLR ligand pattern recognition peptides, and thus inducing higher APC activation. A prominent example to this effect is PROSTVAC, which has utilized plasmid construction to include TLR ligand inserts in the DNA backbone along with the TAA, prostate specific antigen to the viral vector [11].

## 6. Force V: Supplements

### 6.1. Combination with Chemotherapy and Radiotherapy

Anti-cancer chemotherapy targets tumors based on cancer cell’s unique genetics which selectively promotes rapid cell growth over normal terminally differentiated cells. Further, several studies have shown that these anti-cancer drugs can promote reactivation of tumor-specific immune responses by enhancing T-cell infiltration to the tumor microenvironment, and also by making tumor cells more amenable to cytotoxic CD8+ T-cell effector response [89]. Drugs such as cyclophosphamide and gemcitabine have also been known to induce apoptosis of Tregs. A clinical peptide vaccine study with cyclophosphamide has demonstrated that this combination has selectively decreased the number of actively proliferating Treg cells in renal cell carcinoma and ovarian cancers [90]. Therefore, theoretically, chemotherapy and DNA vaccination can be combined to not only because of their rapid cancer cell lysis, but also induce effector immune responses in the tumor microenvironment.

### 6.2. Combination with Immune-Check Point Inhibitors

The signaling mechanisms mediated by co-stimulatory molecules, such as CD28, and OX-40, play an important role in T-cell engagement with antigen presenting cells and its subsequent activation and maturation [91]. Several studies have demonstrated that density of ligands for co-stimulatory molecules in the tumor microenvironment is significantly low and thus leading to T-cell anergy. However, this T-cell unresponsiveness could be overcome by stimulation with agonistic monoclonal antibodies specifically targeted to bind with the co-stimulatory molecules and act as surrogate ligands. Recently, several clinical trials are underway to study the clinical application of these monoclonal antibodies. Along with decreased co-stimulatory molecules, an enhanced expression of co-inhibitory molecules, such as CTLA-4 and PD-1, are known to correlate positively with tumor load and metastasis. Blocking monoclonal antibodies developed against CTLA-4 and PD-1 have already shown promising positive anti-cancer outcomes in clinical trials [92]. A futuristic application of a combination of these immune-checkpoint inhibitors with DNA vaccination would likely enhance the treatment and survival of cancer patients.

## 7. Conclusions

DNA-based non-viral cancer vaccines offer a viable complement to the current anti-cancer therapeutic regimen by inducing robust effector and memory T-cell responses. However, there still remain several challenges requiring more careful optimization of vaccine design to overcome immunosuppressive tumor microenvironment. Therefore, a better understanding of tumor–host immune interactions helps us design more efficient immunotherapeutic strategies. Recent advances in immune checkpoint therapy have revealed an important futuristic combinatorial therapeutic strategy. More research in the identification of specific TAs and neo-antigen could induce strong effector T-cell responses. DNA-based cancer vaccine strategies have promising future applications. Complemented with a better understanding on the TAA selection and immune-checkpoint therapy, they will emerge as viable next-generation therapeutic strategy against cancers.

## Figures and Tables

**Table 1:** List of Human tumor associated antigens potentially applicable for development of DNA vaccines. TAA, tumor-associated antigen.

TAAs	Organs	Reference
NYESO-1	Prostate cancer, bladder cancer, esophagus cancer, non-small cell lung cancer, sarcoma	[12]
HER-2/Neu	Breast	[13]
MAGE-1	Melanoma	[14]
Tyrosinase	Melanoma Leukemia	[15]
MUC1	Breast cancer	[16]
CEA	Colon cancer, lung cancer	[17]
Mam-A	Breast cancer	[18]
hTERT	Melanoma, leukemia, reported several solid organs	[19]
Sialyl-Tn	gastric, colon, breast, lung, oesophageal, prostate and endometrial cancer	[20]
WT1	Renal cancer	[21]
α-FetoProtein	Hepatic cancer	[22]
CA-125	Ovarian cancer	[23]
gp-100	Melanoma	[24]
p53, Ras, Src	reported in multiple cancers	[25]
